# Cardiopulmonary bypass in a pediatric patient with factor XII deficiency

**DOI:** 10.1051/ject/2024021

**Published:** 2024-09-20

**Authors:** Julie M. Fenske, Julie Tinius Juliani, Cynthia Herrington

**Affiliations:** 1 Children's Hospital Los Angeles 4650 Sunset Blvd Los Angeles CA 90027 USA

**Keywords:** Cardiopulmonary bypass, Perfusion, Factor XII, Deficiency, Coagulopathy, Anticoagulation, Pediatrics

## Abstract

The safe use of cardiopulmonary bypass (CPB) relies upon the ability to administer, monitor, and reverse anticoagulation. Although rare, the factor XII deficient patient creates a challenge for the perfusionist due to resultant complications in monitoring anticoagulation. There have been proposed strategies to aid in monitoring anticoagulation in factor XII deficient patients, however, documentation of successful monitoring during CPB is infrequent. With the use of the Hemochron Signature Elite and ACT + cartridges, CPB in a factor XII deficient 8-month-old was completed with predictable and reliable anticoagulation monitoring. This case report explores the current suggestions for factor XII deficiency management with CPB.

## Overview

Factor XII, also known as the Hageman factor, was first identified in 1955 when a patient’s blood failed to demonstrate normal clotting in glass sample tubes [1]. This patient, John Hageman, turned out to be deficient in factor XII and this discovery catalyzed the 1964 “waterfall” model of coagulation by Dr. Davie and Dr. Ratnoff [2]. Factor XII was later described as one of the plasma serine proteases collectively referred to as the contact system responsible for the activation of the intrinsic pathway of coagulation [3]. Factor XII is responsible for multiple processes, including activating factor XI, prekallikrein, and C1 esterase, as well as vasodilation and increased vascular permeability [4]. Understanding developmental hemostasis suggests that most procoagulant factors are low in infancy due primarily to liver immaturity, but reach adult levels by 6 months of age, [5–7].

Factor XII deficiency is normally asymptomatic, and is estimated to occur in approximately one in one million people, [1, 8] although another study found up to 2.3% of the population to be deficient in factor XII [9]. It is most often an autosomal recessive disorder but may also be autosomal dominant [3, 8], and may be heterozygous or more severely homozygous [1]. Deficiency of factor XII is associated with prolonged anticoagulation measures including activated partial thromboplastin time (aPTT) and activated clotting time (ACT), as these tests measure the intrinsic clotting cascade (Conaglen). However, contrary to what is implied with these prolonged anticoagulation measures, factor XII deficient patients do not demonstrate an increase in bleeding time [3, 10, 11].

It is worth mentioning that some studies suggest that there may be an increased risk of thromboembolic complications in factor XII deficient patients [8, 12]. This is thought to be because factor XII is responsible for the conversion of plasminogen into plasmin therefore initiating the fibrinolysis process, however, the relationship between factor XII deficiency and thrombosis is yet to be fully understood [1]. Notably, the life of John Hageman, who was the novel patient afflicted with factor XII deficiency, came to an end after complications from a pulmonary embolism [1].

## Description

An 8-month-old male with a history of VACTERL syndrome, complete tracheal rings #3-8, congenital tracheal stenosis, recurrent tracheal granulomas, and previous cardiac arrest with resuscitation presented for tracheal reconstruction with cardiopulmonary bypass. Coagulation tests performed one month before the operative date revealed a critically low factor XII value of 16.6% (expected range 50–150%) and a high partial thromboplastin time (PTT) of 46 s (expected range 25–35 s) [1]. Prothrombin time (PT) of 13 s and the fibrinogen level of 253 mg/dL were within normal range.

An International Technidyne Corporation (ITC) Hemochron Signature Elite and ACT + cartridges were used for anticoagulation monitoring. Baseline activated clotting time (ACT) was notably normal, at 117 s. Institution protocols call for 100 mg/kg of the antifibrinolytic tranexamic acid (maximum dose of 750 mg) in the cardiopulmonary bypass prime, and 100 mg/kg of tranexamic acid (maximum dose of 750 mg) given intravascularly by anesthesia upon bypass initiation, however, after a preoperative team discussion neither of the antifibrinolytic doses were administered to further inhibit post-operative fibrinolysis in the patient.

**Figure 1:**
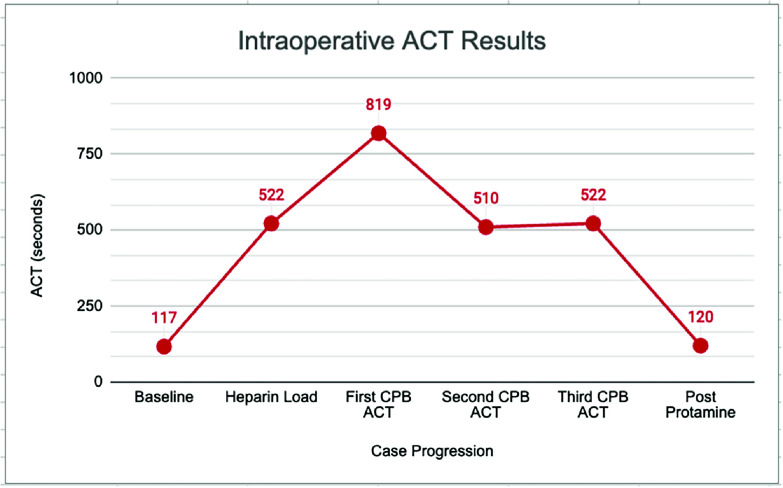
Trend of intraoperative activated clotting time results as provided by the Hemochron.

Sternotomy was made, and upon the surgeon’s request, a loading dose of 2100 units of heparin (300 u/kg per protocol) was administered intravenously by anesthesia. An additional dose of 2000 units of heparin was administered in the cardiopulmonary bypass prime, per institution protocol, as well as the standard dosing of albumin, sodium bicarbonate, solumedrol, mannitol, and cefazolin. Packed red blood cells (125 mL) were also added to the prime to reduce hemodilution upon bypass initiation. Bypass was initiated once the ACT was confirmed to be elevated from baseline and above the target of 400 s, and the bypass run of 65 min was uneventful. Heparin metabolism throughout the pump run was as expected, with ACT results declining from 819 s to 522 s, without additional heparin administration. Heparin was reversed with 24 milligrams of protamine post-bypass (a ratio of 0.59 mg protamine: 100 mg total heparin). Post-protamine ACT was 120 s. Results of the ACT throughout the case are displayed in Figure 1.

Hematocrit trend, lactate trend, and blood gas results throughout the bypass run were unremarkable. The patient was transferred to the CTICU uneventfully. There were no notable postoperative events.

## Comment

Safe implementation of cardiopulmonary bypass is dependent on the ability to monitor, and therefore ensure appropriate anticoagulation. Factor XII deficiency compromises this monitoring as it is one of the contact factors that respond to the activator present in most ACT tests: Celite and/ or kaolin. Celite and kaolin are both widely accepted and used in anticoagulation monitoring, however, kaolin is more reliable for anticoagulation monitoring in patients on the serine protease inhibitor aprotinin [13].

The Hemochron Signature Elite is used to monitor anticoagulation at Children’s Hospital Los Angeles; the Hemochron ACT + cartridges use a mixture of silica, kaolin, and phospholipids as activators (Hemochron manual). With this variety of activators, ACT results are not prolonged as they are with standard Celite or kaolin-only activation. As suggested by the intraoperative ACT results, the Hemochron Signature Elite and ACT + cartridges provided reliable ACT values that were expected before heparin, after heparin, during bypass, and after protamine administration. At the time of writing, the authors have only been able to find one other publication of successful anticoagulation monitoring within factor XII deficiency, using the Hemochron Jr. device and ACT + cartridges [11].

There have been other methods suggested for management of the factor XII deficient patients. One such method is to monitor the anti-Xa concentration of the patient’s blood. Unfortunately, the time required to complete this test is impractical for the application of cardiopulmonary bypass. Monitoring of heparin concentration, as opposed to ACT, has also been suggested using a device such as the Medtronic HMS [14]. However, it must be stated that adequate heparin concentration does not always ensure adequate anticoagulation, such as in the case of antithrombin III deficiency or heparin resistance. Another publication relied on speculation after observing a prolonged (though unreliable) ACT after heparin administration [13, 14]. A more involved method could be to increase factor XII levels through the administration of fresh frozen plasma to increase the factor XII content in the patient. This was discussed as an alternative method if the Hemochron did not prove to be reliable. Gerhardt et al. published a case report demonstrating the titration curve of fresh frozen plasma necessary for increasing factor XII and resulting ACTs within expected ranges. However, it is demonstrated that the subsequent increase in factor XII after FFP administration is short-term [3, 15].

Further, it is critical to consider the use of antifibrinolytics in cardiac surgery with factor XII deficient patients. Factor XII deficiency impairs fibrinolysis, as activated factor XII usually activates pre-kallikrein to kallikrein furthering fibrinolysis. Without the normal degree of fibrinolysis, dosing an antifibrinolytic should be reconsidered [4]. If available, viscoelastic testing such as thromboelastography (TEG) or rotational thromboelastometry (ROTEM) should be considered for the factor XII deficient patient, as these tests provide timely insight into clot initiation, clot formation, and fibrinolysis. A 2022 publication by Stelmach et al. found factor XII deficient patients (n = 20) to have markedly higher coagulation times and clot formation times, though no marked difference in maximum clot formation or maximum lysis [16] At the time of this case, there was no access to viscoelastic technology for patient blood sample analysis.

With adequate preparation and knowledge, anticoagulation can be managed adequately for cardiopulmonary bypass to be reliably performed. There have been suggestions within the literature regarding strategies to monitor anticoagulation in factor XII deficient patients, however, the authors find that the safest and most reliable method is the use of an array of anticoagulant activators, such as with the Hemochron ACT + cartridges for the Hemochron Signature Elite. Future research should be completed to investigate the mechanism by which the ACT + activator allows for reliable anticoagulation monitoring in factor XII deficient patients and validate the reliability of this monitoring method in different levels of factor XII deficiency.

## Data Availability

All available data are incorporated into the article.

## References

[1] Azaad MA, Quirong Z, Yongping Li. Factor XII (Hageman factor) deficiency: a very rare coagulation disorder. Open J Blood Dis. 2015;5(4):39–42.

[2] Caen J, Wu Q. Hageman factor, platelets and polyphosphates: early history and recent connection. J Thromb Hemost. 2010;8(8):1670–1674. 10.1111/j.1538-7836.2010.03893.x PMC2965785 20456750

[3] Conaglen PJ, Akowuah E, Sanjay T, Atkinson V. Implications for cardiac surgery in patients with factor XII deficiency. Ann Thorac Surg. 2010;89(2):625–626. 20103363 10.1016/j.athoracsur.2009.07.042

[4] Schmaier A. The elusive physiologic role of factor XII. J Clin Invest. 2008;118(9):3006–3009. 18725991 10.1172/JCI36617 PMC2518076

[5] Andrew M, Patsy V, Johnston M, et al. Maturation of the hemostatic system during childhood. Blood. 1992;80(8):1998–2005. 1391957

[6] Andrew M, Paes B, Milner R, et al. Development of the human coagulation system in the full-term infant. Blood. 1987;70(1):165–172. 3593964

[7] Hanmod S, Jesudas R, Kulkarni R, Chitlur M. Neonatal hemostatic disorders: issues and challenges. Semin Thromb Hemost. 2016;42(7):741–751. 10.1055/s-0036-1593415. 27706533

[8] Chaudhry LA, El-Sadek WYM, Chaudhry GA, et al. Factor XII (Hageman factor) deficiency: a rare harbinger of life threatening complications. Pan Afr Med J. 2019;33:39. 31384354 10.11604/pamj.2019.33.39.18117 PMC6658145

[9] Halbmayer WM, Haushofer A, Schön R, et al. The prevalence of moderate and severe FXII (Hageman factor) deficiency among the normal population: evaluation of the incidence of FXII deficiency among 300 healthy blood donors. Throm Haemost. 1994;71(1):068–072. 8165648

[10] Gerhardt MA, Greenberg CD, Slaughter TF, et al. Factor XII deficiency and cardiopulmonary bypass: use of a novel modification of the activated clotting time to monitor anticoagulation. Anesthesiology. 1997;87:990–992. 9357906 10.1097/00000542-199710000-00038

[11] Erkinaro T, Moilanen J, Lahtinen J, et al. The standard point-of-care Hemochron Jr. ACT + test in monitoring heparin administration for cardiopulmonary bypass in severe factor XII deficiency. J Cardiothorac Vasc Anesth. 2022;36(7):2031–2034. 34130893 10.1053/j.jvca.2021.05.021

[12] Regling K, Ramiz S, Chitlur MB. Factor XII deficiency in ECMO patients. Blood. 2019;134(1):4965.

[13] De Vries A, Lansink-Hartgring AO, Fernhout FJ, et al. Activated clotting time in cardiac surgery: should Celite or kaolin be used? Interact CardioVasc Thorac Surg. 2017;24(4):549–554. 28108578 10.1093/icvts/ivw435

[14] Uppal V, Rosin M. Factor XII deficiency and cardiopulmonary bypass. J Extra Corpor Technol. 2014;46(3):254–257. 26357792 PMC4566835

[15] Cronbaugh RD, Fuller LA, Miller SD, et al. Cardiopulmonary bypass in a patient with factor XII deficiency. J Extra Corpor Technol. 2014;46(3):251–253. 26357791 PMC4566834

[16] Stelmach P, Nowak W, Robak M, Krzemińska E, Tybura-Sawicka M, Chojnowski K, Treliński J. In seeking diagnostic tool for laboratory monitoring of FXII-targeting agents, could assessment of rotational thromboelastometry (rotem) in patients with factor XII deficiency be useful? Acta Haematol Polon. 2022;53(4):267–272. 10.5603/ahp.a2022.0034.

